# Supplementary material for: RIM‐binding proteins recruit BK‐channels to presynaptic release sites adjacent to voltage‐gated Ca2+‐channels
Source: EMBO J. 2018 Jul 2;37(16):e98637. doi: 10.15252/embj.201798637 (PMC6092624; doi:10.15252/embj.201798637)
Supplement: Supplementary file 1 — Appendix [file EMBJ-37-e98637-s001.pdf]

Appendix for Sclip et al.,
"RIM-Binding Proteins Recruit BK-Channels to Presynaptic Release Sites Adjacent to Voltage-Gated Ca^{2+}-Channels"

Table of contents

Appendix tables……………………………………………………………page 1
Appendix figures and figure legends………………………………………… page 5
Appendix methods……………………………………………………………...page 15
Appendix references…………………………………………………………page 22

APPENDIX TABLES

Appendix Table S1: **List of confirmed yeast two-hybrid preys isolated with RBP2 baits** (related to Figure 1)

GENE	PROTEIN NAME	UNIPROT ID	CLONES	
Cacna1	calcium channel, voltage-dependent, N type, alpha 1B subunit	Q02294	10	3-8-12-22-34-45-61-62-66-76
Lrrtm2	leucine rich repeat transmembrane neuronal 2	D4A7P2	6	46-55-69-80-94-47
Ppp2r5d	protein phosphatase 2, regulatory subunit B', delta		6	7-42-36-93-95-102
Mab21l2	mab-21-like 2	D4ACZ1	5	34-43-43b-44-52
KCNMA1	potassium large conductance calcium-activated channel alpha member 1	Q62976	4	37-38-30-32
Zfp710	zinc finger protein 710 (Zfp710)	D4A0K8	4	54-49-21-98
Mocs3	molybdenum cofactor synthesis 3	D4A8L5	3	18-37-53
Plec	plectin	P30427	3	27-35-36
Ankrd44	ankyrin repeat domain 44	F1LZH9	2	80-81
Cep170	centrosomal protein 170		2	24-103
LOC361646	K04F10.2 (LOC361646)	F1LX85	2	3-4
Sox11	SRY (sex determining region Y)-box 11	P0C1G9	2	11-39
Abhd4	abhydrolase domain containing 4 (Abhd4)	D3ZAW4	1	70
Arih1	ariadne ubiquitin-conjugating enzyme E2 binding protein homolog 1	D3ZXL1	1	100
CCT3	S288c Cct3p	Q6P502	1	79
CCT6	S288c Cct6p		1	41
Celf2	Elav-like family member 2	Q792H5	1	79
CEP192	centrosomal protein 192kDa		1	48
CEP290	centrosomal protein 290kDa	A0A0G2K929	1	17
Ciz1	CDKN1A interacting zinc finger protein 1	F1LV60	1	41
DCAF7	DDB1 and CUL4 associated factor 7	B2RZ68	1	178
Doc2b	Double C2-like domain-containing protein beta	P70610	1	149
Dpysl2	dihydropyrimidinase-like 2	P47942	1	40
Eif4g2	eukaryotic translation initiation factor 4, gamma 2	F1LN59	1	14
EPHA8	EPH receptor A8	P29321	1	9
Fat1	FAT atypical cadherin 1	A0A0G2K5L1	1	87
Flrt2	fibronectin leucine rich transmembrane protein 2	D3ZTV3	1	25

Frs2	fibroblast growth factor receptor substrate 2	A0A0G2JZZ5	1	26
Gga1	Golgi associated, gamma adaptin ear containing, ARF binding protein 1	Q5FVF3	1	96
GIGYF1	acetyl-CoA acetyltransferase 1 (Acat1) or GRB10 interacting GYF protein 1	D3ZQJ3	1	5
Gltscr1	GLTSCR1-like	D4A240	1	31
Gnaq	guanine nucleotide binding protein, alpha q polypeptide	P82471	1	86
Insr	insulin receptor	P15127	1	10
Jund	jun D proto-oncogene	P52909	1	11
Klc1	kinesin light chain 1	P37285	1	101
Lars	leucyl-tRNA synthetase	Q5PPJ6	1	62
LOC102553329	uncharacterized LOC102553329		1	82
Lphn1	latrophilin 1	O88917	1	77
LRRC4B	leucine rich repeat containing 4B	P0CC10	1	17
Maged1	melanoma antigen, family D, 1	Q9ES73	1	66
Mark4	MAP/microtubule affinity-regulating kinase 4	D4A6T9	1	28
Mbd1	methyl-CpG binding domain protein 1	Q66HB8	1	32
Mdm4	transformed mouse 3T3 cell double minute 4	Q5XIN1	1	73
MGC79934	uncharacterized protein MGC79934		1	26
Mycbp2	MYC binding protein 2	D4A2D3	1	41
NOB1	NIN1/RPN12 binding protein 1 homolog	Q6VEU1	1	75
Npr2	natriuretic peptide receptor 2	P16067	1	31
Nrd1	nardilysin 1	P47245	1	83
Nsmf	NMDA receptor synaptonuclear signaling and neuronal migration factor	Q9EPI6	1	22
Ntm	neurotrimin	Q62718	1	2
Pnn	desmosome associated protein	D3ZAY8	1	99
Prdm10	PR domain containing 10	D3ZQ79	1	151
Prr12	proline rich 12	D4A6M5	1	47
R3HCC1L	R3H domain and coiled-coil containing 1-like	A0A0G2JZM0	1	52
Sept5	septin 5 (Sept5)	Q9JJM9	1	51
Sestd1	SEC14 and spectrin domains 1	B5DFL9	1	11
Slc45a1	solute carrier family 45, member 1	Q8K4S3	1	15
Snrpg	small nuclear ribonucleoprotein polypeptide G		1	82
St3gal2	ST3 beta-galactoside alpha-2,3-sialyltransferase 2	Q11205	1	46
Sv2b	synaptic vesicle protein 2B	Q63564	1	52
Synj2bp	synaptojanin 2 binding protein	Q9WVJ4	1	33
Tead2	TEA domain family member 2	B5DF69	1	38
Tet1	tet methylcytosine dioxygenase 1	F1LUQ3	1	54
Thra	thyroid hormone receptor alpha	P63059	1	81
Tnip2	TNFAIP3 interacting protein 2	G3V7R6	1	15
Trappc10	trafficking protein particle complex 10	F1MAQ4	1	61
WDTC1	WD and tetratricopeptide repeats 1	D4A7Z0	1	43
Zbtb44	zinc finger and BTB domain containing 44	Q3SWU4	1	6
Zdbf2	zinc finger, DBF-type containing 2	D4A582	1	29
Zfp238	zinc finger protein 238	Q9JKY3	1	16
Zfp516	zinc finger protein 516	A0A0G2K242	1	34

Zmym2	zinc finger, MYM-type 2	Q9CU65	1	4

---

*Preys isolated using RBP2 (247-859) are shown in black, preys isolated using RBP2 (1-859) in blue, and preys isolated using RBP2 (247-1068) in red (see Figure S1)

Appendix Table S2: **Subcellular localization of reconfirmed preys based on literature analyses** (related to Figure 1)

Uniprot ID	GENE	Plasma membrane	Membrane	Cytoplasm	Nucleus	Synapse	Ion channel	Calcium associated
D3ZAW4	Abhd4							
F1LZH9	Ankrd44							
D3ZXL1	Arih1			●				
Q02294	Cacna1b	●	●				●	●
Q6P502	CCT3	●		●	●			
	CCT6							
Q792H5	Celf2			●	●			
	Cep192							
A0A0G2K929	Cep290		●	●	●			
F1LV60	Ciz1				●			
B2RZ68	Dcaf7			●				
P70610	Doc2b	●	●	●		●		●
P47942	Dpysl2		●	●		●		
F1LN59	Eif4g2					●		
P29321	EPHA8	●	●			●		
A0A0G2K5L1	Fat1	●	●		●			●
D3ZTV3	Flrt2	●	●	●		●		
A0A0G2JZZ5	Frs2	●		●				
Q5FVF3	Gga1		●					
D3ZQJ3	GIGYF1							
D4A240	Gltscr1							
P82471	Gnaq	●	●	●	●			
P15127	Insr	●	●	●	●	●		
P52909	Jund				●			
Q62976	Kcnma1	●	●				●	
P37285	Klc1		●	●		●		
Q5PPJ6	Lars			●	●			
	LOC102553329							
F1LX85	LOC361646	●			●			
O88917	Lphn1	●				●		
P0CC10	LRRC4B	●	●	●		●		
D4A7P2	Lrrtm2	●	●			●		
D4ACZ1	Mab21l2				●			
Q9ES73	Maged1	●	●	●	●			
D4A6T9	Mark4							
Q66HB8	Mbd1			●	●			
Q5XIN1	Mdm4				●			
	MGC79934							
D4A8L5	Mocs3			●				
D4A2D3	Mycbp2		●					
Q6VEU1	NOB1				●			
P16067	Npr2	●	●					
P47245	Nrd1							
Q9EPI6	Nsmf	●	●	●	●			●
Q62718	Ntm	●	●			●		
P30427	Plec			●				
D3ZAY8	Pnn		●					
	Ppp2r5d							
D3ZQ79	Prdm10							
D4A6M5	Prr12							
A0A0G2JZM0	R3HCC1L							
Q9JJM9	Sept5	●		●		●		
B5DFL9	Sestd1					●		●
Q8K4S3	Slc45a1		●					
B5DEP7	Snrpg							
P0C1G9	Sox11			●	●			
Q11205	St3gal2		●					
Q63564	Sv2b		●			●		
Q9WVJ4	Synj2bp		●					
B5DF69	Tead2				●			
F1LUQ3	Tet1							
P63059	Thra			●	●			
G3V7R6	Tnip2			●	●			
F1MAQ4	Trappc10			●		●		
D4A7Z0	Wdtc1			●	●			
Q3SWU4	Zbtb44				●			
D4A582	Zdbf2							
A0A0G2K242	Zfp516				●			
D4A0K8	Zfp710				●			
Q9CU65	Zmym2			●	●			

APPENDIX FIGURES and FIGURE LEGENDS

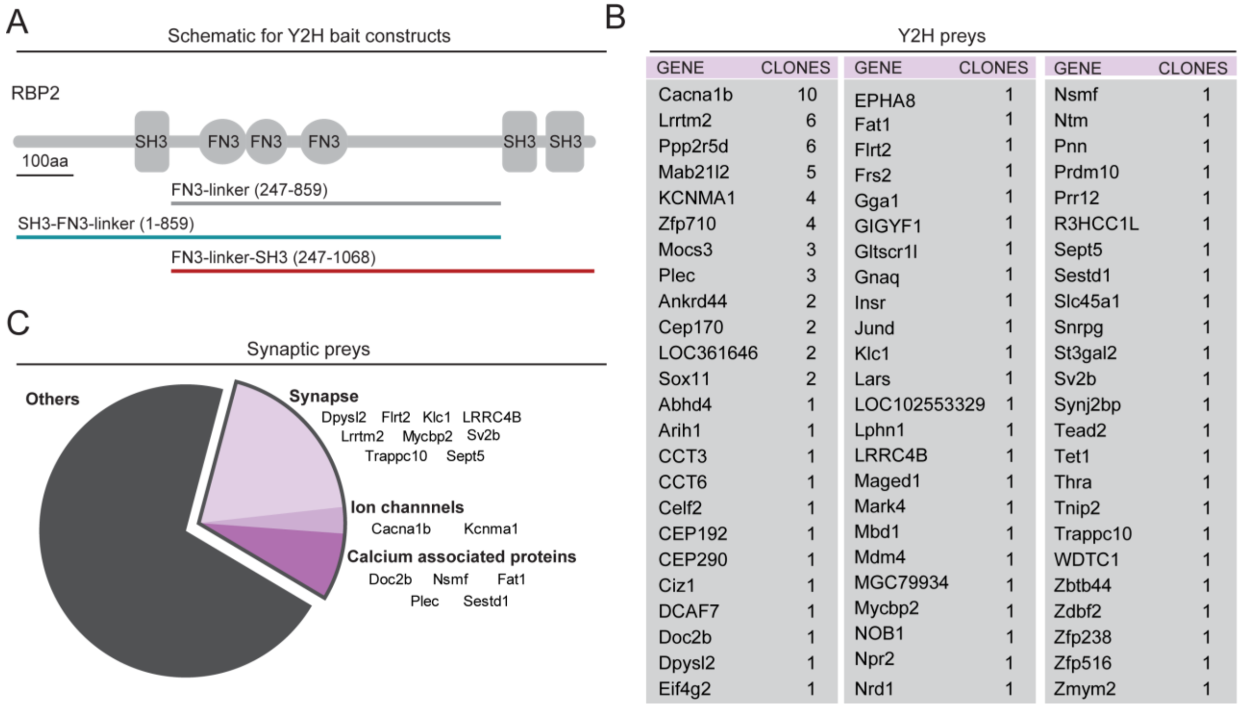

GENE	CLONES	GENE	CLONES	GENE	CLONES
Cacna1b	10	EPHA8	1	Nsmf	1
Lrrtm2	6	Fat1	1	Ntm	1
Ppp2r5d	6	Flrt2	1	Pnn	1
Mab21l2	5	Frs2	1	Prdm10	1
KCNMA1	4	Gga1	1	Prr12	1
Zfp710	4	GIGYF1	1	R3HCC1L	1
Mocs3	3	Gltscr1l	1	Sept5	1
Plec	3	Gnaq	1	Sestd1	1
Ankrd44	2	Insr	1	Slc45a1	1
Cep170	2	Jund	1	Snrpg	1
LOC361646	2	Klc1	1	St3gal2	1
Sox11	2	Lars	1	Sv2b	1
Abhd4	1	LOC102553329	1	Synj2bp	1
Arih1	1	Lphn1	1	Tead2	1
CCT3	1	LRRC4B	1	Tet1	1
CCT6	1	Maged1	1	Thra	1
Celf2	1	Mark4	1	Tnip2	1
CEP192	1	Mbd1	1	Trappc10	1
CEP290	1	Mdm4	1	WDTC1	1
Ciz1	1	MGC79934	1	Zbtb44	1
DCAF7	1	Mycbp2	1	Zdbf2	1
Doc2b	1	NOB1	1	Zfp238	1
Dpysl2	1	Npr2	1	Zfp516	1
Eif4g2	1	Nrd1	1	Zmym2	1

Appendix Figure S1: **Yeast two-hybrid screening** (related to Figure 1)

A, Schematic of RBP2 domain structure highlighting the location of the 3 baits used for yeast two-hybrid (Y2H) screening: (1) a construct covering aa 247-859 (containing the FN3 domains and the linker region), (2) a construct covering aa 1-859 of RBP2 (N-terminal SH3-FN3-linker), and (3) a construct covering aa 247-1068 (FN3-linker-C-terminal SH3 domains).

B, List of preys recovered and reconfirmed, with number of clones observed.

C, Pie chart showing preys associated to synapses (GO: 0045202, 0030424, 0098793, 0016192, 0042734, 0060076, 0043679, 0008021, 0006906), related to calcium binding or regulation (GO: 0035584, 0005509, 0048306, 0034704) or annotated as voltage gated ion channels (GO: 0005244)

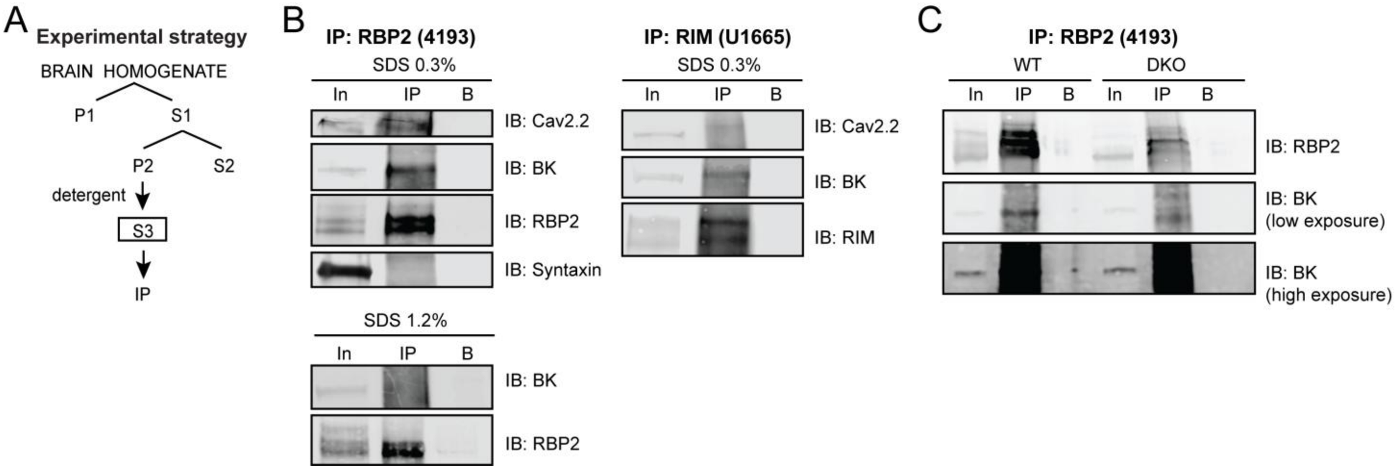

Appendix Figure S2: **Co-immunoprecipitation of endogenous RBP2, RIMs, and BKα** (related to Figures 1-3)

A, Schematic of brain lysate preparation for immunoprecipitation. After homogenization, brain lysates were spun at 800 g to remove the nuclei (P1). The supernatant (S1) was centrifuged at 13000 rpm for 15 min to collect the membrane enriched fraction (P2) and discard the cytosolic fraction (S2). The membrane enriched fraction (P2) was then incubated with buffers containing different detergents and subsequently centrifuged at 13000 rpm for 15 min. The supernatant was then collected and used as starting material for immunoprecipitations (IPs).

B, Immunoblotting analyses of IPs of RBP2 or RIMs performed in mouse brain homogenates solubilized with the indicated concentrations of SDS. Immunoprecipitations were performed with the RBP2 antibody 4193 or the RIM antibody U1565. Immunoblots (IB) were performed with the following antibodies: RBP2, 4193; BKα, APC-021 (Alomone); Cav2.2; ACC-002 (Alomone); syntaxin-1, HPC-1 (SySy). See also Figure 1D. Abbreviations: In = input (1% of total protein), IP = immunoprecipitation, B = controls using preimmune serum, IB = immunoblot. See also Figure 1G.

C, Immunoblotting analyses of RBP2 IPs performed in mouse brain homogenates from wild type (WT, left) or constitutive RBP1,2 double knock-out mice (DKO, right) as control. Immunoprecipitations were performed with the RBP2 antibody 4193 after solubilisation of the proteins with 1% Triton X-100. Immunoblots were performed with the following antibodies: RBP2, 4193; and BKα, APC-021 (Alomone). Abbreviations: In = input (1% of total protein), IP = immunoprecipitation, B = controls using preimmune serum, IB = immunoblot. Note that signal for BKα was detected when immunoprecipitations were performed with homogenates from WT mice, but failed to be detected when samples from constitutive RBP1,2 double KO mice were used, despite similar levels of BKα in WT and DKO inputs.

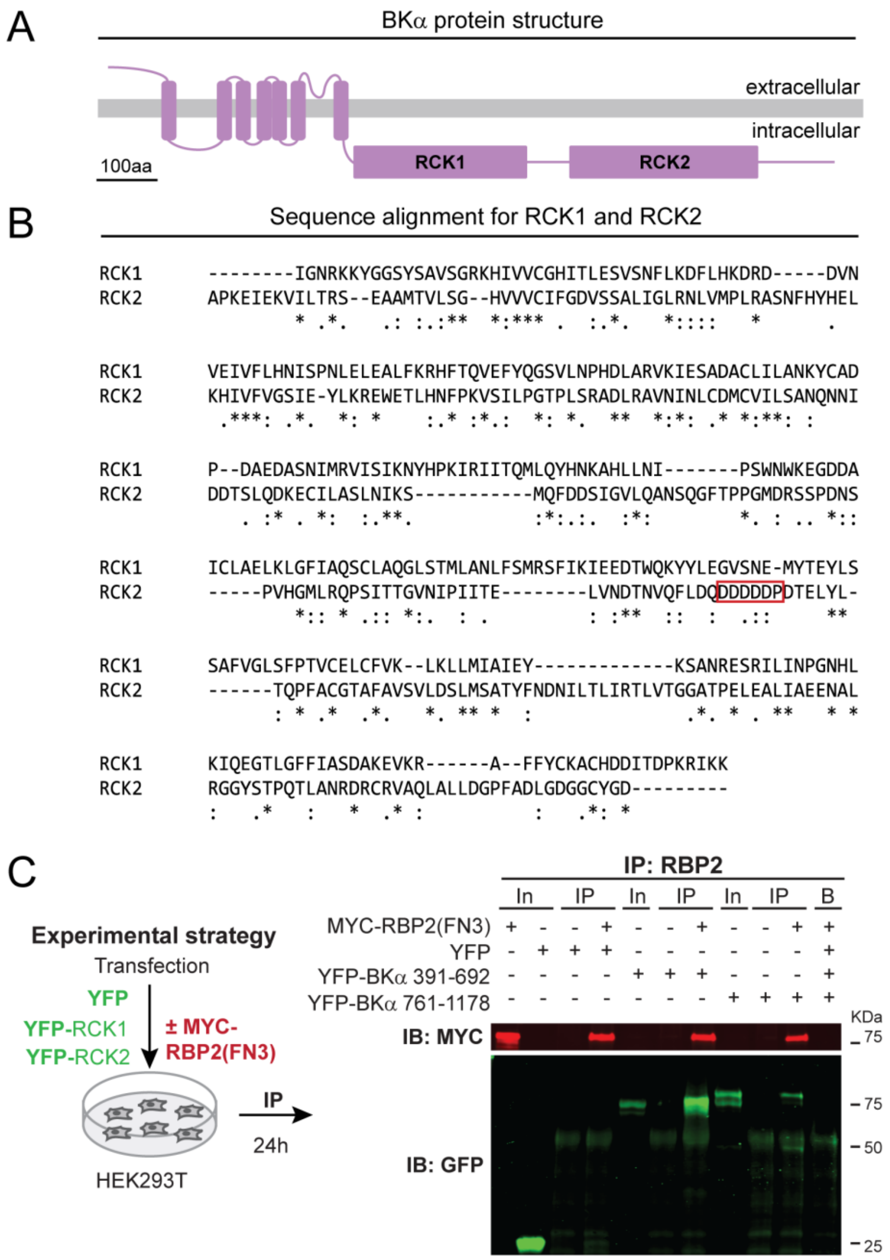

Appendix Figure S3: **Location and homology of RCK domains in BKα channel subunit** (related to Figure 3)

A, Domain structure of the BKα-protein containing 7 transmembrane regions and two intracellular RCK domains (RCK1 and RCK2).

B, Alignment of the human RCK1 and RCK2 domains, showing high homology of sequence ("*", identical amino acids; ":" for conserved substitutions; "." for semi-conserved substitutions). The presumptive Ca^{2+}-binding site in the RCK2 is highlighted by a red box.

C, Experimental strategy for validating the interaction of the FN3-domain of RBP2 with the RCK1 and RCK2-domains of BKα (left), and immunoblot analyses co-immunoprecipitation experiments (right). Co-immunoprecipitations were performed on transfected HEK293T cell

expressing the myc-tagged RBP2 (FN3 domains) in combination with YFP, YFP-tagged BKα (RCK1) or YFP-tagged BKα (RCK2) proteins. In the immunoblots on the right, RBP2-immunoprecipitations of cell lysates from transfected HEK293T cells expressing either myc-tagged RBP2 (FN3) alone or together with YFP or the indicated YFP-tagged BKα RCK domains. Input fractions (In; 1% of total) or immunoprecipitates (IP) were analysed by immunoblotting (IB) with antibodies to myc (red) or GFP (green, recognizes YFP). Abbreviations: In = input (1% of total protein), IP = immunoprecipitation, B = controls using preimmune serum, IB = immunoblot.

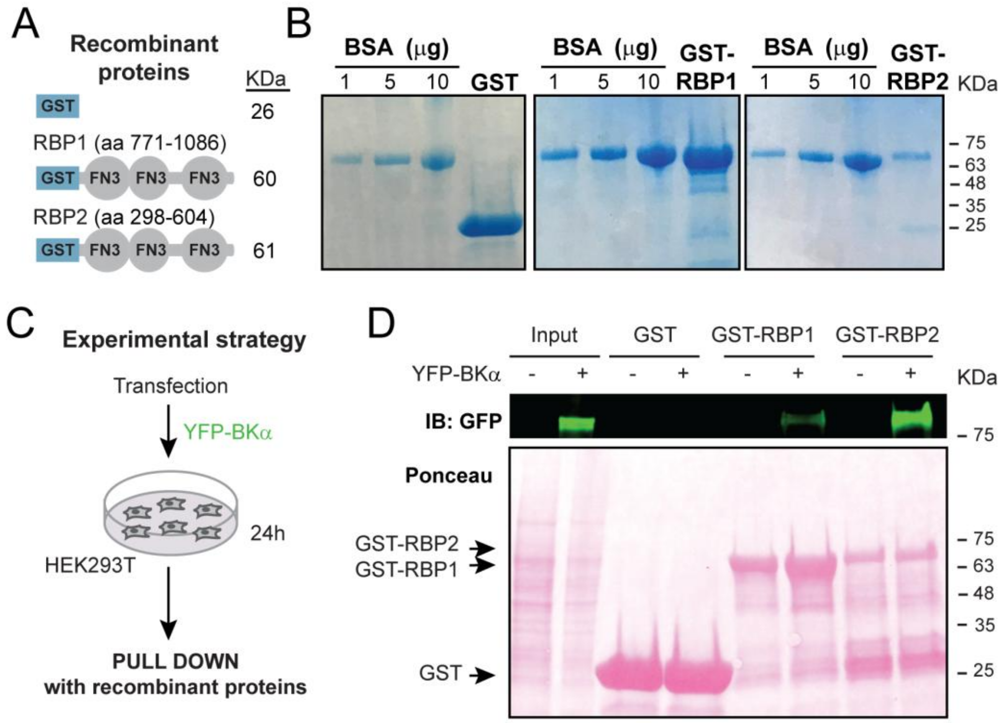

Appendix Figure S4: **Pull down of BKα channels with recombinant FN3-domains of RBP1 and RBP2 (related to Figures 1-3)**

A, Schematic of the GST-fused recombinant proteins purified for the pull down experiment: GST alone as negative control, GST-fused RBP1 (aa 771-1086), and GST-fused RBP2 (aa 298-604), corresponding to the FN3 domains.

B, Comassie staining for 10 µl of purified proteins, obtained from 1l bacterial cultures (beads were resuspended in 2 ml of washing buffer) and compared to different BSA concentrations (1, 5 and 10 µg).

C, Schematic of the pull down experimental strategy. YFP-BKα was expressed in HEK293T cells. 24h after transfection, YFP-BKα expressing cells as well as not transfected cells were lysed and incubated with the GST-fused recombinant proteins described in A, B.

D, Pull down experiment as outlined in C, showing in the upper part immunoblot for GFP (T3743), to detect pulled down YFP-BKα, and on the bottom Ponceau staining, to visualize the GST-fused recombinant proteins. Abbreviation: IB= immunoblot.

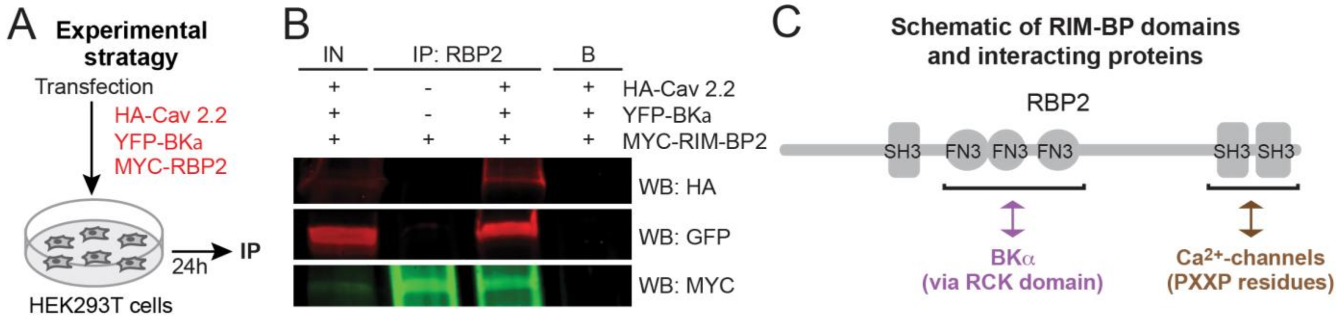

Appendix Figure S5: **RBP2 forms a simultaneous complex with BKα and the Ca^{2+}-channel Cav2.2** (related to Figures 1-3)

A, Strategy for reconstituting the RBP2-BKα-Cav2.2 complex in transfected HEK293T cells and for analysing the complex by immunoprecipitations. The proteins listed in red were expressed as epitope-tagged full-length proteins.

B, Co-immunoprecipitation of Cav2.2 and BKα with RBP2 co-expressed in HEK293T cells as outlined in C. Abbreviations: In = input, IP = immunoprecipitation, B = controls using preimmune serum, IB = immunoblot.

C, Domain structure of RBP2 and location of BKα and Cav2.2 binding sites.

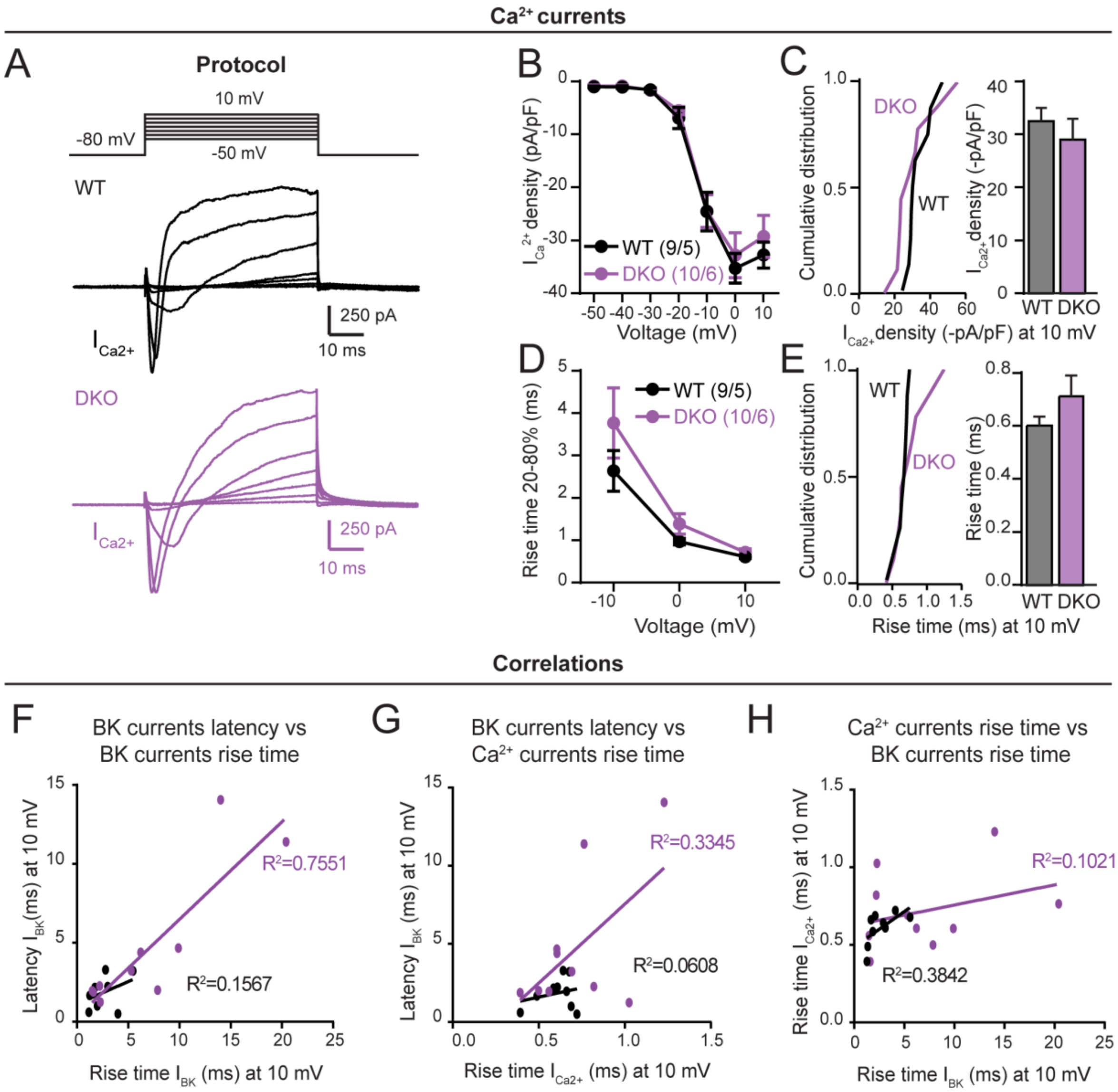

Appendix Figure S6: **Loss of RBPs does not alter presynaptic Ca^{2+}-currents** (related to Figure 5)

A, Simultaneous recordings of calcium (inward) and potassium (outward) currents at the Calyx of Held. Top: presynaptic depolarization protocol. Bottom: sample traces for a control (black) and a RBP DKO (magenta) terminal.

B, Ca^{2+}-current density as a function of voltage in RBP WT and DKO terminals.

C, Cumulative distribution of current density at maximal voltage (+10 mV) (left) and bar graph showing the averaged Ca^{2+}-current density at 10 mV (right) in WT and DKO Calyx terminals.

D, Ca^{2+}-current 20-80% rise time as a function of voltage in WT and DKO terminals.

E, Left: cumulative distribution of Ca^{2+}-current rise time triggered by depolarization to +10 mV. Right: bar graph showing the averaged I_{Ca2+} density at 10 mV.

F-H, Correlation graphs showing the relationship between the latency and rise time of BK currents recorded at 10 mV (F), between the latency of BK currents and the rise time of calcium currents (G), and between the rise time of BK currents and calcium currents (H) in individual experiments.

Data are expressed as means ± SEM. Significance was calculated for B-D with Two-way ANOVA for repetitive measurements, with WT=9/5 and DKO=10/6, n.s. $p>0.05$ and for C-E (bar graph) using the Student t-test, with WT=9/5 and DKO=10/6, n.s. $p>0.05$.

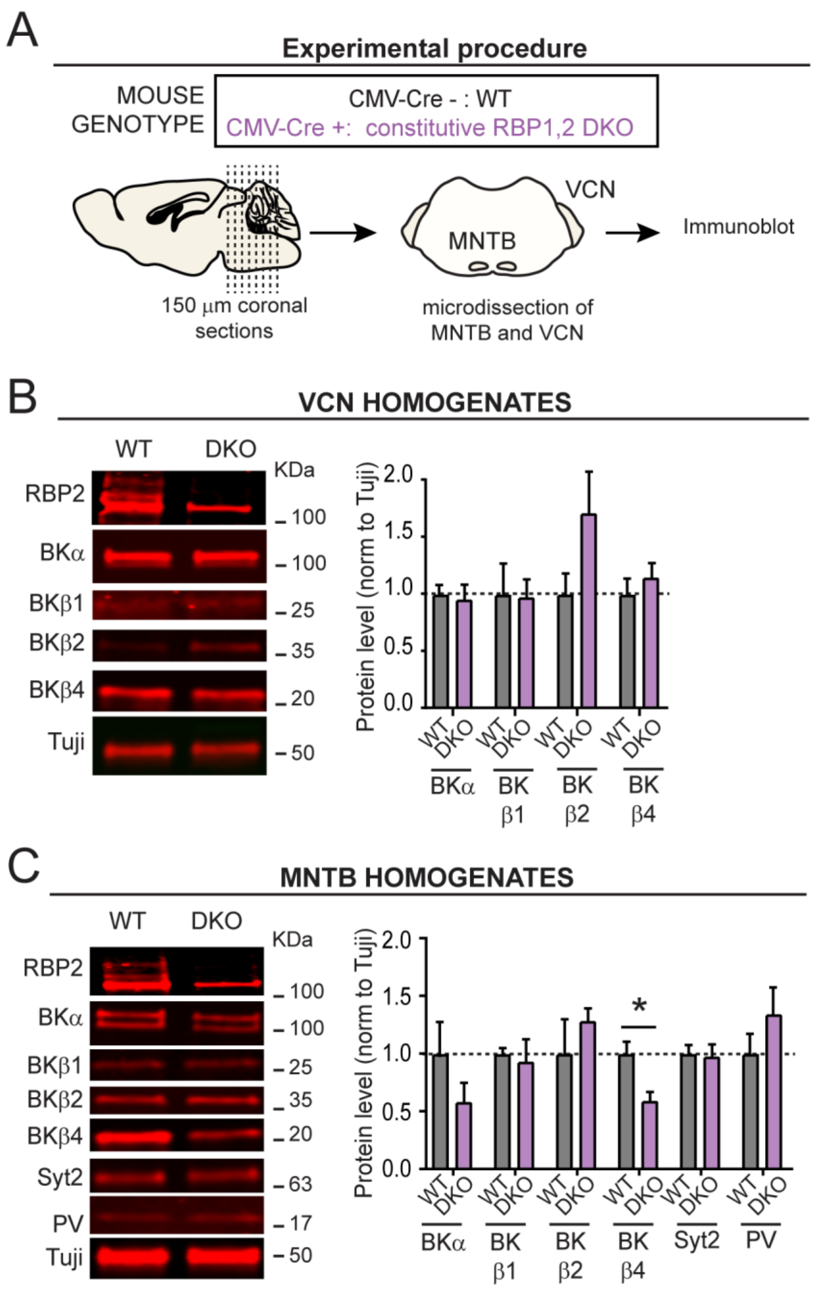

Appendix Figure S7: **Expression levels of BK channel subunits in samples from the MNTB and VCN micro-dissected from littermate wild-type and RBP1/2 DKO mice** (related to Fig. 7)

A, Experimental strategy outlining the dissection protocol. Wild type (WT) or constitutive RBP1,2 DKO mice (DKO) were used for this set of experiments. Brain were removed and 150 µm thick coronal slices were obtained on a vibratome. The medial nucleus of the trapezoid body (MNTB) and the ventral cochlear nucleus (VCN) were micro-dissected from each slice, homogenized and used for immunoblot experiments, as showed in B and C.

B, Representative images and relative quantification for RBP2 and BK channel subunits in VCN homogenates from WT and constitutive RBP1,2 DKO mice.

B, Representative images and relative quantification for RBP2 and BK channel subunits in MNTB homogenates from WT and constitutive RBP1,2 DKO mice. Syt2 and PV were used as marker for the MNTB.

Data are expressed as means ± SEM. Significance was calculated for B-D using the Student t-test, with WT (n=6) and DKO (n=6). *= p>0.05.

APPENDIX METHODS

Expression vectors. For yeast two-hybrid experiments: pBTM116-RBP2 aa1-859, pBTM116-RBP2 aa247-859, and pBTM116-RBP2 aa 247-1068 were cloned from full-length rat RBP2 (Wang et al, 2000) into the EcoRI/SalI sites. For overexpression experiments in HEK293T cells: pCMV5-myc-RBP2 aa 1-1068, pCMV5-myc-RBP2 aa 247-859, pCMV5-myc-RBP2 aa 1-1068(Δ247-859) were cloned from full-length rat RBP2 (Wang et al, 2000) into the EcoRI/XbaI sites. BK alpha partial clone was isolated from the rat brain library in the yeast-two-hybrid screening and re-cloned into the pCMV5-YFP vector (Addgene) using BglII/EcoRI sites. Full-length BK alpha (human) was obtained from Harvard plasmid database. pCMV5-YFP-BKα (RCK1) (aa 391-692), and pCMV5-YFP-BKα (RCK2) (aa 761-1178) were cloned from the Harvard plasmid into BclII/SalI sites. pCMV5-YFP vector was used to express YFP. pCMV5-YFP-RIM1α construct (Kaeser et al, 2011) was used as positive control. For voltage gated calcium channel overexpression protocols pCMV5-HA-Cav2.2 (rat) was used (Acuna et al, 2015). For expression of recombinant proteins: pGEX-RBP1-FN3 (aa 771-1086) and pGEX-RBP2 –FN3 (aa 298-604) were cloned into NcoI- SacI sites.

Yeast two-hybrid experiments. Yeast two-hybrid screens of a rat brain cDNA library in pVP16-3 were performed using bait vectors encoding for RBP2. Different domains of RBP2 (construct #1: residues 1-859, containing the N-terminus SH3 domain and the FN3 domains; construct #2: residues 247-859 which contains only the FN3 domains; and construct #3: residues 247-1068, containing the FN3 domains and the c terminal SH3 domains) were cloned into the pBTM116 plasmid, expressing Trp for selection. The constructs were transformed into L40 strain with LiAc and selected on complete supplement mixture (CSM) plates (Yeast nitrogen base, 10X dropout solution, 2% agar, 2% glucose) without Trp. L40 strains containing the bait plasmids were grown in large scale, transformed with the library and plated into CSM plates lacking Trp, Leu and His for the screening. 3-amino-1,2,4-triazole (3-AT) was added to the plates at the concentration of 50 mM in order to prevent auto-activation. 1:100, 1:1000 and 1:10.000 dilution of the transformed yeast were plated onto CSM plates lacking Trp and Leu to test the transformation efficiency and assess the number of cloned screened. We screened 4.4 million colonies for pBTM116-RBP2 (aa 247-859), 3 million colonies for pBTM116-RBP2 (aa 1-859), and 6 million colonies for pBTM116- RBP2 (aa247-1068). Positive clones were picked and yeast DNA was isolated as described by (Singh & Weil, 2002). Prey plasmids were then recovered in E. coli, and sequenced with sense primer V1 (GTT TAC CGA TGC CCT TGG) and if necessary with anti-sense primer YW01156 (CGT TGT AAA ACG GCC). 95 positive clones were reconfirmed for Y2H screening with pBTM116-RBP2 (aa 247-859) as bait, 93 for pBTM116-RBP2 (aa 1-859), 58 for pBTM116- RBP2 (aa247-1068). Growth of prey clones onto CSM plates lacking Trp, Leu and His was retested and clones that were not able to grow were excluded from the final list. Preys were annotated with Uniprot ID using the PANTHER database (www.pantherdb.org), as reported in Table S1 and

clustered for subcellular localization and association to synapses using quick Go web service (https://www.ebi.ac.uk/QuickGO/), see Figure S1, Table S2.

Sequence alignments. Sequence alignment of human RCK1 and RCK2 domains (Figure S3) have been performed with the ClustalW2 multiple sequence alignment program (http://www.ebi.ac.uk/Tools/msa/clustalw2/).

Immunoprecipitations. For immunoprecipitation experiments in HEK293T cells, cells were plated into 60 mm culture dishes and transfected with calcium phosphate. 24 h after transfection cells were lysed in lysis buffer containing (in mM): 20 HEPES, pH 7.5, 100 NaCl, 4 KCl, 2 $MgCl_2$, 2 $CaCl_2$, 0.1 PMSF, 1% Triton X-100 and complete protease inhibitors (Roche). Lysates were incubated for 20 min on ice and then centrifuged at 14000 rpm for 10 min to remove the insoluble fraction. The supernatant was collected and precleared for 1.5 h at 4°C with sepharose beads. Precleared lysates were used for immunoprecipitation. Lysates were pre-incubated for 30 min with the primary antibody (GFP, T3743, Rabbit, homemade; or RBP2, 4193, Rabbit, homemade), before adding sepharose beads. After 12h, beads were washed 5 times in lysis buffer and resuspended in Laemmli buffer, boiled for 10 min at 99°C and analyzed by Immunoblot.

For immunoprecipitation from brain lysates, BL6 mice were anesthetized and decapitated. Brains were collected and homogenized in a sucrose buffer containing 0.32M sucrose. Nuclei were removed by centrifugation at 800xg for 10 min at 4°C. The supernatant was collected and centrifuged at 14000 rpm for 15 min at 4°C. The pellet containing the membrane fraction was then incubated with different detergent to solubilized the protein contained in the membrane: 1% Triton X-100 for 30 min at 4°C, 1% NP-40 for 30 min at 4°C or SDS (0.3% or 1.2%) at RT for min followed by treatment with 1.2% cold Triton X-100 (4:1 volume) at 4°C (Geumann et al, 2010). Solubilized proteins were collected by centrifugation at 14000 rpm for 15 min and used for IP. Immunoprecipitations were performed following the protocol previously described for HEK293T cells, using antibodies against RBP2 (4193) or RIM (U1565).

Co-localization experiments. For imaging experiments, HEK293T cells were plated into 24 well plates and transfected with calcium phosphate. 24 h after transfection cells were detached with 10 mM EDTA in PBS and spotted onto glass coverslips coated with matrigel. Cells were allowed to attach to the coverslip for 30 min and then fixed in 4% PFA for 15 min. Cells were permeabilized with 0.1% Triton X-100 in PBS for 3 min, blocked in 5% NGS in PBS for 1h and incubated o/n at 4°C with anti-myc antibody (9E10, Hybridoma Bank), 1:500 in blocking solution. Cells were then washed three times in PBS, incubated for 1h at RT with Alexa 546 secondary antibody (Invitrogen), 1:500 in blocking solution. Cells were washed three times in PBS, mounted on superfrost slides and covered with mounting media containing dapi (Southern biotech). Serial confocal z-stack images (1 µm intervals at 1024 x 1024 resolution) were acquired using a Nikon confocal microscope (A1Rsi) with a 60x oil objective (PlanApo, NA1.4). For every section, we calculated the colocalization between YFP and dapi by averaging the Pearson's coefficient, using the

Nikon analysis software. Pearson's coefficients were then normalized to the control condition.

Preparation of recombinant proteins. GST-tagged FN3 domains of RBP1 and RBP2 were expressed in Rosetta™ 2(DE3) bacteria. Bacteria were grown to OD 0.5 and GST-fused protein expression was induced with 0.1 mM IPTG for 2h at 37°C for GST alone, for 12 hours at room temperature for RBP1 and for 12 hours at 18°C for RBP2. Bacteria were harvested by centrifugation for 20 min at 4,000 x g , 4°C, and pellets were resuspended in STE buffer (100 mM NaCl, 1 mM EDTA, 10 mM Tris-HCl pH 8.0) supplemented with 0.5 mg/ml lysozyme, 1% Triton X-100, 2mM DTT, 0.1 mM PMSF and protease inhibitor cocktail, and incubated for 30 min at 4°C. Cells were lysed by one freeze/thaw cycle followed by sonication (3× 15 pulses, 50% output). Insoluble material was removed by centrifugation for 30 min at 7,000xg, 4°C. Proteins were affinity-purified using glutathione sepharose beads (GE Healthcare) incubation for 1 hour at 4°C, beads were washed 3x with STE with 500mM NaCl. Proteins were stored at 4°C in STE containing 0.1mM PMSF and protease inhibitor cocktail. Protein concentration was assessed by Western blot followed by Comassie staining. 20 µl of beads were loaded on the gel and compared to 1-5-10 µg of BSA.

Pull-down assays. 5 µg of purified GST- proteins associated to gluthatione beads (GE Healthcare) were incubated with protein extracts from HEK293T cells expressing YFP-BKα for 30 min at 4°C. Beads were then washed 3x times with the lysis buffer (20 mM Tris-HCl pH 7.5, 100 mM NaCl, 4 mM KCl, 2 mM $MgCl_2$, 2 mM $CaCl_2$) and resuspended in sample buffer followed by immunoblotting against GFP, which recognized the YFP tag. Protein signals were visualized by fluorescently labelled secondary antibodies on Odyssey CLx Infrared Imager and Odyssey software (LI-COR Biosciences). GST-tagged proteins were visualized with Ponceau staining.

RBP1,2 double KO mice and genotyping protocol. Conditional RBP1 and RBP-2 KO mice were generated as described by Acuna et al. (Acuna et al, 2015; Acuna et al, 2016). Mice were genotyped by PCR using a standard program: 95°C 2', (94°C 30'', 60°C 30'', 72°C 1' x 35 cycles), 72°C 7'. The following oligonucleotide primers were used for genotyping RBP1 and RBP2 conditional mouse lines: CA873 TTAGATCATGGAGTGAGGTGTGC and CA874 CCCAGCTCTTCAGCATCTACC to genotype RBP1 mice (the expected band size is 171 for the WT allele and 328 for the mutant allele); CA881 TGTTATGTCTCTCCCTGATTGC and CA882 AAGGAGTCCCAGTGCATAGG CCCAGCTCTTCAGCATCTACC to genotype RBP2 mice (the expected band size is 257 for the WT allele and 413 for the mutant allele).

RBP1 and RBP2 conditional mice were crossed to a CMV-Cre mouse line (stock number 006054, Jackson's labs) to generate constitutive RBP1 or RBP2 knockout mice (Acuna et al, 2015; Schwenk et al, 1995). These lines were subsequentially bred together to generate homozygous RBP1 and RBP2 double knockout mice. Mice were genotyped by PCR using a standard program: 95°C 2', (94°C 30'', 60°C 30'', 72°C 1' x 35 cycles), 72°C

7'. The following primers were used: CA875 TGGACATAGCAGAGGTCGTCC and CA876 CCCAGCTCTTCAGCATCTACC (expected size: 585 bp for the WT allele and 349 for the KO) and CA873 TTAGATCATGGAGTGAGGTGTGC and CA874 CCCAGCTCTTCAGCATCTACC (expected size: 171 for the WT allele and 328 for the mutant) to genotype RBP1 mice; CA879 CAATGGCAGACTTCATGAGG and CA882 AAGGAGTCCCAGTGCATAGG 351 (expected size: 585 bp for the WT allele and 351 bp for the KO) and CA881 TGTTATGTCTCTCCCTGATTGC and CA882 AAGGAGTCCCAGTGCATAGG (expected size: 257 for the WT allele and 413 for the mutant) to genotype RBP2 mice.

To specifically remove RBP1 and RBP2 at the Calyx of Held terminals, we crossed RBP1 and RBP2 conditional double knockout mice to a tissue-specific Cre line, the Krox20-Cre line (Acuna et al, 2015; Voiculescu et al, 2000). These mice express Cre under the control of the Krox20 promoter in the anterior ventral cochlear nucleus (aVCN), whose neurons give rise to the calyx of Held terminals (Acuna et al, 2015). Breeding pairs composed by a Krox20-Cre positive homozygotes RBP1/2 conditional mouse and a Krox20-Cre negative homozygotes RBP1/2 conditional mouse were set up, so that 50% of the offspring was expected to carry the Cre allele (which allows removal of RBP1 and RBP2 from the Calyx of Held synapses) while 50% of the offspring was negative for Cre and therefore used as wild type control (referred as WT in the figures).

Preparation of brain lysates. Intact brain samples were removed from constitutive RBP1 and RBP2 double knockout (DKO) mice and wild type (WT) mice. Samples were homogenized in 0.32 M ice-cold sucrose buffer containing (in mM): 1 HEPES, 1 $MgCl_2$, 1 EDTA, 1 $NaHCO_3$, 0.1 PMSF, and 1% Triton X-100 at pH 7.4, in the presence of a complete set of protease inhibitors (Complete; Roche Diagnostics, Basel, Switzerland). For medial nucleus of the trapezoid body (MNTB) and ventral cochlear nucleus (VCN) homogenates, intact brains were first removed and trimmed and 150 μm-thick coronal slices were cut with a Leica vibratome in cold ACSF. MNTB and VCN were then micro-dissected and homogenized in 50 µl of sucrose buffer. Protein concentration was quantified using the Bradford Assay (Bio-Rad Protein Assay 500-0006, München, Germany). 10-40 μg of brain homogenates (respectively for MNTB or VCN homogenates and total brain homogenates) were separated and analysed by Immunoblot.

Immunoblotting experiments. Immunoprecipitation or brain samples were prepared as described above and separated by SDS-PAGE using 4-20% mini protean TGX precast gels (Bio-Rad). Protein were transferred onto nitrocellulose membranes for 10 min at 2.5 V using the Trans-blot turbo transfer system (Bio-Rad). Membranes were blocked in Tris-buffered saline (5% no-fat milk powder, 0.1% Tween20) for 1h at room temperature. Primary antibodies were diluted in the same buffer and incubated overnight at 4°C.The following antibodies were used at 1:1000 dilution: RBP2 (4193), Myc (9E10, Developmental Studies Hybridoma Bank), GFP (T3743 or JL-8, Clontech), KCNMA1 (APC-021/APC-107, Alomone) KCNMB1 (APC-036, Alomone), KCNMB2 (APC-034,

Alomone), KCNMB4 (APC-061, Alomone), Cav2.2 (ACC-002), RIM (U1565), Syntaxin (HPC-1), Synaptotagmin 2 (znp-1, ZFIN), Parvalbumin (P3088, Sigma), Tubulin (T2200, Sigma). Combinations of the following IRDye secondary antibodies were used (1:10.000 dilution): IRDye 800CW donkey anti mouse (926-32212), IRDye 680LT donkey anti mouse (926-68022), IRDye 800CW donkey anti rabbit (926-32213), IRDye 680LT donkey anti rabbit (926-68023), from LI-COR. Pseudo colours were then applied to the signals. Detection of the signal was obtained by Odyssey CLx imaging systems (LI-COR). Quantification was performed with Image Studio 5.2 free software.

Immunohistochemistry. Mice were anesthetized, perfused with phosphate buffered saline (PBS) and subsequently fixed with 4% paraformaldehyde (PFA) in PBS via a perfusion pump (2 ml/min). The brain of each mouse was dissected out, post-fixed in 4% PFA for 1h at 4°C and cryoprotected in 30% sucrose for 36 h at 4°C. 30 µm coronal sections were cut with a cryostat (Leica CM1050). Sections were washed with PBS, incubated in blocking solution (0.3% Triton X-100 and 5% goat serum in PBS) for 1h at RT under gentle agitation, and incubated for 48 h at 4°C with primary antibodies diluted in blocking solution (anti-vGluT1, 1:500, guinea pig, AB5905 Millipore; alpha subunit of BK channel, 1:200, rabbit, APC-107, Alomone). Sections were washed 4 times (15 min each time) in PBS, incubated with secondary antibodies (1:500, Alexa 488, 545, 633, Invitrogen) at 4°C overnight, and washed 4 times (15 min each time) again in PBS. Sections were then mounted on superfrost slides and covered with mounting media (Southern biotech). Serial confocal z-stack images (1.5 µm intervals at 2048 x 2048 resolution) were acquired using a Nikon confocal microscope (A1Rsi) with a 60x oil objective (PlanApo, NA1.4). All acquisition parameters were kept constant among different conditions within experiments. On average, 3 brain sections from a single animal were stained and imaged (n≥8 animals per condition). For every section, we calculated the area of presynaptic BK channel colocalizing with vGlut1, using the Nikon analysis software. We then normalized the BK area to the area of the vGlut1 signal and finally averaged the results obtained from single sections of the same animal.

Measurement of BK channel properties in HEK293T cells. HEK293T cells were plated on 24-well plates coated with matrigel and transfected after 24 h (60% confluence) using lipofectamine (Invitrogen, 0.7 µl/well) with full-length human BK channel alpha subunit alone or in combination with RBP2 constructs. pCMV-YFP was cotransfected in all conditions to identify transfected cells. Electrophysiological recordings were performed 24 h after transfection. On the day of recording, cells were trypsin-dissociated, and plated on matrigel-coated coverslips. After 1h recovery, coverslips were transferred into a recording chamber mounted on an upright microscope. Whole-cell voltage-clamp recordings were performed at room temperature using ~3 mΩ glass pipettes. Cell responses were sampled at 10 KHz and leak-corrected using a P/4 protocol. The following bath solution was used (in mM): 144 NaCl, 5.8 KCl, 0.9 $MgCl_2$, 1.3 $CaCl_2$, 0.1 NaH_2PO_4, 5.6 glucose, 10 HEPES-KOH, 2.5 4-aminopyridine (4-AP), pH~7.4. The internal solution contained (in mM): 135 KCl, 3.5 $MgCl_2$, 2 $ATPNa_2$, 0.5 EGTA, 5.04 $CaCl_2$, 5 HEPES-NaOH pH 7.2, resulting in

a free Ca^{2+}-concentration of ~10 μM, which was estimated using the maxchelator website (http://maxchelator.stanford.edu/webmaxc/webmaxcE.htm). Cells were held at -80 mV holding potentials unless otherwise indicated. Summarized data display G-V curves which were fitted with a Boltzmann equation, using IGOR Pro software, to obtain half-maximum activation voltages as well as the G-V curve slope.

Preparation of acute brain slices for electrophysiology. Acute brainstem slices containing the medial nucleus of the trapezoid body (MNTB, containing the calyx of Held synapses) were prepared from 10-12 days old RBP1,2-Krox20- Cre+ and Cre- mice as previously described. Isofluorane-anethesized mice were decapitated, their brain removed and trimmed, and the midbrain/brainstem placed in an ice-cold oxygenated (95% O_2, 5% CO_2) cutting solution that contained (in mM): 125 NaCl, 2.5 KCl, 3 $MgCl_2$, 0.1 $CaCl_2$, 25 glucose, 1.25 NaH_2PO_4, 0.4 ascorbic acid, 3 myo-inositol, 2 Na-pyruvate, and 25 $NaHCO_3$ (pH 7.4). Around 150 μm-thick slices that included the medial nucleus of the trapezoid body (MNTB) were cut with a Leica vibratome (VT1200S) and kept for 30 minutes in warm (32 ºC) oxygenated ACSF solution containing (in mM): 125 NaCl, 2.5 KCl, 1 $MgCl_2$, 2 $CaCl_2$, 25 glucose, 1.25 NaH_2PO_4, 0.4 ascorbic acid, 3 myo-inositol, 2 Na-pyruvate, and 25 $NaHCO_3$ pH 7.4. The brain slices were then moved to holding chamber filled with oxygenated ACSF at room temperature for 1 h for recovery. Then, MNTB-containing slices were placed into a PM-6D recording chamber (Warner Instruments, Inc) mounted onto an Axioskop FS-2 upright microscope (Zeiss). The microscope was equipped with DIC and fluorescence filters, and a Lambda DG4 fluorescent lamp connected to the back port of the microscope via an optic fiber. Slices were maintained at ~23-25°C via a dual-T344 temperature controller (Warner Instruments). Calyx-containing slices were continuously perfused with normal oxygenated ACSF (at about 1 ml/min perfusion rate). Presynaptic elements of calyx of Held synapses were identified under DIC optics and then patched under visual guidance. Electrical signals were recorded at 25 kHz with a two channel Axoclamp 700B amplifier (Axon Instruments), digitalized with a Digidata 1440 digitizer (Molecular devices) that was in turn controlled by Clampex 10.1 (Molecular Devices).

Electrophysiological recordings. Presynaptic BK currents were studied using direct whole-cell voltage-clamp recordings from Calyx terminals. BK currents were triggered by depolarizing pulses from -80 mV holding potentials to -50, -40, -20, -0, -10, 0, and +10 mV for 50 ms, as described by Nakamura et al. (Nakamura & Takahashi, 2007). For this, we used a pipette solution that contained (in mM): 97.5 potassium gluconate, 32.5 KCl, 10 HEPES, 1 $MgCl_2$, 12 Na_2 phosphocreatine, 2 ATP-Mg and 0.5 GTP-Na (305 mOsm l−1, pH 7.3 adjusted with KOH; final K^+ concentration, 143.5 mM). The internal solution was supplemented with the Ca^{2+}-chelator EGTA at the concentration of 0.2 mM (for Figures 5-6) and 10 mM (for Figure 7). The external solution (standard ACSF) contained (in mM): 125 NaCl, 2.5 KCl, 25 $NaHCO_3$, 1.25 NaH_2PO_4, 2$CaCl_2$, 1 $MgCl_2$, 10 glucose, 3 myo-inositol, 2 sodium pyruvate, 0.5 ascorbic acid, 1 μM Tetrodotoxin (TTX) and 2.5 mM 4-aminopyridine (4-AP), pH 7.4 when bubbled with 95% O_2 and 5% CO_2. Under these conditions, pure BK currents could not be pharmacologically isolated due to the presence

of: 1) slow voltage-gated K^+ currents, which are insensitive to 4-AP, 2) inward calcium currents, which are required for BK activation. To quantify pure BK currents, we perfused slices (at the rate of 1 ml/min) with the selective BK-channel blocker Iberiotoxin (IbTX; 200nM), and digitally subtracted the responses before and after application of IbTX.

Data analysis and statistics. Electrophysiological data were analyzed using Clampfit 10.4 (Molecular Devices) or Igor Pro 4.07 (WaveMetrics, Lake Oswego, OR). Statistical analysis was done using GraphPad Prism software.

REAGENT OR RESOURCE	SOURCE	IDENTIFIER
**Antibodies**		
Anti-Cav2.2	Alomone	ACC-002, RRID: AB_2039766
Anti-GFP	Südhof lab	T3743, RRID: AB_2636878
Anti-GFP	Clontech	JL-8, RRID: AB_10013427
Anti-KCNMA1	Alomone	APC-021, RRID: AB_2313725
Anti-KCNMA1	Alomone	APC-107, RRID: AB_2040091
Anti-KCNMB1	Alomone	APC-036, RRID: AB_2040095
Anti-KCNMB2	Alomone	APC-034, RRID: AB_2039955
Anti-KCNMB4	Alomone	APC-061, RRID: AB_2040097
Anti-myc	Developmental Studies Hybridoma Bank	9E10, RRID: CVCL_G671
Anti-Parvalbumin	Sigma	P3088, RRID:AB_477329
Anti-RBP2	Südhof lab	4193, RRID:AB_2617050
Anti-RIM	Südhof lab	U1565, RRID: AB_262133
Anti-Syntaxin	SySy.com	HPC-1
Anti-Synaptotagmin 2	ZFIN	Znp-1, RRID:AB_10013783
Anti-Tubulin	Sigma	T2200, RRID: AB_621847
Anti-vGluT1	Millipore	AB5905, RRID: AB_2301751
Alexa fluor 546, goat anti mouse IgG	Invitrogen	A-11003, RRID: AB_2534071
Alexa fluor 488, goat anti guinea pig IgG	Invitrogen	A-11073 RRID: AB_2534117
Alexa fluor 633, goat anti mouse IgG	Invitrogen	A-21050 RRID: AB_2535718
IRDye 680LT donkey anti mouse	Licor	926-68022, RRID: AB_621848
IRDye 680LT donkey anti rabbit	Licor	926-68023, RRID: AB_10706167
IRDye 800CW donkey anti mouse	Licor	926-32212, RRID: AB_10715072
IRDye 800CW donkey anti rabbit	Licor	926-32213, RRID: AB_621848
**Chemicals**		
Iberiotoxin (IbTx)	Alomone	STI-400
Tetrodotoxin citrate	Tocris	1069
4-aminopyridine	Sigma	275875

Lithium acetate	Sigma	517992
3-Amino-1,2,4-triazole (3-AT)	Sigma	A-8056
**Experimental models**		
Mouse: RBP1	Südhof lab	MGI: 5510577
Mouse: RBP2	Südhof lab	MGI: 5510579
Mouse: ACTB-Flpe	The Jackson laboratory	RRID: IMSR_JAX:005703
Mouse: Krox20 cre	The Jackson laboratory	RRID: IMSR_JAX:025744
Mouse: CMV-cre	The Jackson laboratory	RRID: IMSR_JAX:006054
**Software and algorithms**		
Clampfit	Molecular device	N/A
Igor Pro	Wavemetrics Inc	RRID:SCR_000325
FIJI	NIH	RRID:SCR_002285
pClamp	Molecular device	RRID:SCR_011323
Prism	Graphpad Inc	RRID: SCR_002798
Image Studio Lite	Licor	RRID: SCR_014211
NIS-elements	Nikon	RRID:SCR_002776
Panther database		http://pantherdb.org/
Quick go		https://www.ebi.ac.uk/QuickGO/

APPENDIX REFERENCES

Acuna C, Liu X, Gonzalez A, Sudhof TC (2015) RIM-BPs Mediate Tight Coupling of Action Potentials to Ca(2+)-Triggered Neurotransmitter Release. *Neuron* **87:** 1234-1247

Acuna C, Liu X, Sudhof TC (2016) How to Make an Active Zone: Unexpected Universal Functional Redundancy between RIMs and RIM-BPs. *Neuron* **91:** 792-807

Geumann C, Gronborg M, Hellwig M, Martens H, Jahn R (2010) A sandwich enzyme-linked immunosorbent assay for the quantification of insoluble membrane and scaffold proteins. *Analytical biochemistry* **402:** 161-169

Kaeser PS, Deng L, Wang Y, Dulubova I, Liu X, Rizo J, Sudhof TC (2011) RIM proteins tether Ca2+ channels to presynaptic active zones via a direct PDZ-domain interaction. *Cell* **144:** 282-295

Nakamura Y, Takahashi T (2007) Developmental changes in potassium currents at the rat calyx of Held presynaptic terminal. *The Journal of physiology* **581:** 1101-1112

Schwenk F, Baron U, Rajewsky K (1995) A cre-transgenic mouse strain for the ubiquitous deletion of loxP-flanked gene segments including deletion in germ cells. *Nucleic acids research* **23:** 5080-5081

Singh MV, Weil PA (2002) A method for plasmid purification directly from yeast. *Analytical biochemistry* **307:** 13-17

Voiculescu O, Charnay P, Schneider-Maunoury S (2000) Expression pattern of a Krox-20/Cre knock-in allele in the developing hindbrain, bones, and peripheral nervous system. *Genesis* **26:** 123-126

Wang Y, Sugita S, Sudhof TC (2000) The RIM/NIM family of neuronal C2 domain proteins. Interactions with Rab3 and a new class of Src homology 3 domain proteins. *The Journal of biological chemistry* **275:** 20033-20044
