# Supplementary figures and images for: RIM‐binding proteins recruit BK‐channels to presynaptic release sites adjacent to voltage‐gated Ca2+‐channels
Source: EMBO J. 2018 Jul 2;37(16):e98637. doi: 10.15252/embj.201798637 (PMC6092624; doi:10.15252/embj.201798637)

# S2C

**IP: RBP2 (4193)**

WT

DKO

In

IP

B

In

IP

B

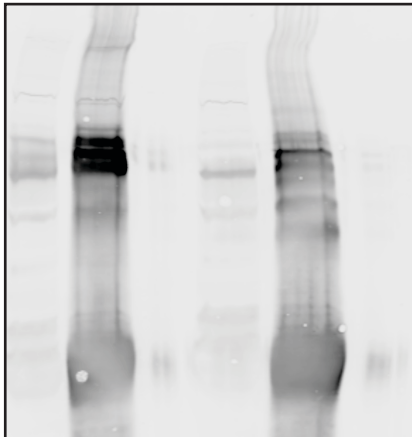

IB: RBP2

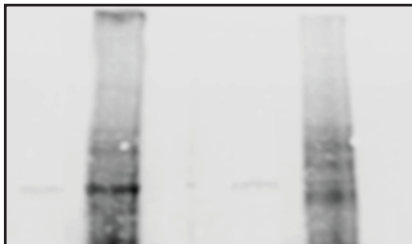

IB: BK $\alpha$   
(low exposure)

Supplement: Supplementary file 2 — Source Data for Appendix [file EMBJ-37-e98637-s002.zip › Sclip_RBP-supplementary_figure_2C.pdf]
